# Personality-Driven Variations in Fitness App Affordance Actualization Among Adults: Quantitative Survey Study

**DOI:** 10.2196/72691

**Published:** 2025-09-12

**Authors:** Moayad Alshawmar, Bengisu Tulu, E Vance Wilson, Adrienne Hall-Phillips

**Affiliations:** 1 College of Business, Imam Mohammad Ibn Saud Islamic University (IMSIU), Al Thoumamah Rd, Riyadh, 11564, Saudi Arabia; 2 Business School, Worcester Polytechnic Institute, Worcester, MA, United States

**Keywords:** fitness apps, personality traits, affordance actualization, user interaction, mHealth app, mobile health

## Abstract

**Background:**

Fitness apps aim to advance individuals’ health and wellness by encouraging consistent healthy habits. Despite their widespread use, sustaining user engagement remains a challenge. Research studies on fitness apps have identified app affordances as one of the key factors that influence user engagement. Some affordances, such as exercise guidance and activity status updates, are shown to support users in achieving their health goals if the users actualize them. However, these affordances need to be actualized by the users to seize these benefits. While identifying these app affordances can deepen our insight into user-app interactions, the impact of personality traits on the actualization of these affordances remains underexplored.

**Objective:**

This study aims to examine the influence of personality traits on the actualization of fitness app affordances.

**Methods:**

Building on affordance actualization theory and the Big Five personality framework, we hypothesized about certain personality traits influencing the actualization of certain app affordances. We tested these hypotheses using a survey of adult Fitbit app (Google LLC) users (N=442). We used validated measures from the literature to assess these variables. We analyzed the survey data using covariance-based structural equation modeling.

**Results:**

Our findings reveal distinct affordance actualization patterns based on users’ personality traits. Users with the conscientious personality trait primarily actualize the updating affordance (β=0.136, *P*=.01), while the influence of the conscientious trait on actualization of rewards (β=–0.154, *P*=.06), competing (β=−0.118, *P*=.18), comparing (β=–0.084, *P*=.33), reminding (β=−0.060, *P*=.44), or guidance (β=−0.006, *P*=.95) affordances was not significant. The openness to experience trait showed a significant positive effect on actualization of updating affordances (β=0.227, *P*=.001), but did not significantly influence actualization of searching (β=−0.172, *P*=.11), watching others (β=−0.077, *P*=.50), or guidance (β=−0.005, *P*=.96) affordances. Users with the agreeableness trait actualized comparison (β=0.213, *P*=.02), guidance (β=0.259, *P*=.003), and encouragement (β=0.244, *P*=.01) affordances, while the effect of the agreeableness trait on actualization of watching others was not significant (β=0.143, *P*=.13). Extravert users actualized recognition (β=0.191, *P*<.001), self-presentation (β=0.165, *P*=.002), and watching others (β=0.167, *P*=.003) affordances, but did not actualize updating affordances (β=0.001, *P*=.98). Finally, a lower emotional stability trait did not significantly influence any of the hypothesized affordances, with nonsignificant effects on guidance (β=−0.083, *P*=.30), reminding (β=−0.093, *P*=.21), and updates (β=−0.036, *P*=.49).

**Conclusions:**

Our study shows that certain personality traits are associated with the actualization of specific affordances. These findings underscore the need to tailor fitness app affordances to individual differences, rather than relying on a one-size-fits-all approach. Designing fitness app functionality that aligns with various personality traits may promote deeper and more sustained user engagement. Further research is needed to investigate the relationship between personality traits and app affordance actualization.

## Introduction

Fitness apps encourage users to engage in regular physical activity by offering personalized guidance, progress tracking, and social support [1]. When used consistently, these apps can enhance physical and mental well-being, reduce the risk of chronic disease, and improve memory [2,3]. Despite these benefits, many users discontinue use shortly after downloading [4,5]. Understanding the factors that influence sustained engagement is therefore critical.

One way to explain this discontinuation is by examining the affordances that users perceive and actualize, opportunities for action provided by the app. Studies have identified common affordances in fitness apps, such as exercise guidance, social comparison, rewards, watching others, and reminders [6-10]. These affordances are shaped by users’ goals and how they interact with the app, rather than by design alone. As Leonardi [11] notes, individuals’ goals determine what they perceive technology features as enabling. Strong et al [12] distinguish affordances as “potentials for action” and actualization of affordances as “actions taken by individuals to realize those potentials” through the use of technology. Research shows that these goals influence the affordances users actualize; for example, those with extrinsic exercise goals may avoid social affordances [9], while older users may value health progress updates [13].

While users’ goals influence which affordances they actualize, these goals themselves may be shaped by deeper, stable individual characteristics, such as their personality traits. Personality theory suggests that life pursuits are expressions of underlying dispositions [14]. In the personality traits literature, the Five-Factor Model, also known as Big Five traits, is the most widely accepted among the many approaches to identifying personality traits [15]. The Big Five traits characterize human personality into 5 major characteristics while accepting that an individual will tend to have some of the traits more than others [16]. The Big Five traits, agreeableness, conscientiousness, extraversion, neuroticism, and openness capture broad individual differences in tendencies such as cooperation, organization, sociability, emotional stability, and curiosity [17].

Prior work links these traits to technology use behaviors, including fitness apps [15,18-20], yet it remains unclear whether personality influences which affordances users actualize. Accordingly, this study examines how personality traits influence the actualization of specific fitness app affordances. We hypothesize that variations in the Big Five traits will lead to differences in which affordances users actualize.

People high in conscientiousness are organized, efficient, and achievement-oriented [21]. They excel at following physical activity guidelines [22] and perform tasks more effectively with reminder technologies [15]. In fitness apps, conscientious users rate competitive strategies as strong motivators [23]. Given their drive to accomplish goals, we hypothesize (H1) that conscientious Fitbit (Google LLC) users will actualize affordances related to achievement and exercise effectiveness, such as guidance, reminding, updating, competing, rewards, and comparing, allowing them to complete exercises correctly and on time and to track their progress.

People high in agreeableness are cooperative, compassionate, and warm [21]. They thrive in collaborative settings [24] and engage with technologies that support teamwork [25]. In fitness apps, they are drawn to features that foster social connection, such as viewing others’ progress, challenging others, and exercising in live workout sessions [19,25,26]. Agreeable people are also caring in nature and expect others to repay such care [27]. Therefore, we hypothesize (H2) that because agreeable people are more likely to engage in collaborative work and care more about others, they will actualize comparing, guidelines, watching others, and encouragement affordances.

Emotional stability reflects individuals’ control over their emotions [28]. Those with lower stability tend to be anxious and insecure [28] and often report lower satisfaction with work and technology use [16,27,29-31]. However, when faced with health concerns, they may become highly motivated to improve their health [32-34]. One study reported that such individuals smoked fewer cigarettes, drank less alcohol, and quit smoking immediately when they faced negative health conditions [35]. Another study reported a positive impact of users’ having lower emotional stability on how they rate the fitness apps’ usefulness [36]. Given their focus on managing health, we hypothesize (H3) that Fitbit users with lower emotional stability will actualize affordances such as updating, guidance, and reminding that help them monitor health status, perform exercises correctly, and stay on schedule.

People high in openness are imaginative, curious, and receptive to new ideas [21]. They embrace change, explore novel activities such as mobile health apps, and are intrinsically motivated by enjoyment rather than external rewards [37-41]. Given their curiosity and desire to avoid exercise errors, we hypothesize (H4) that open Fitbit users will actualize affordances that support exploration and accurate performance, such as searching, watching others, guidance, and updating.

People high in extraversion are sociable, outgoing, and energetic [38]. Their desire for social connection and maintaining a positive image drives engagement in interactive activities [25], both in real life and on social media [42,43]. In fitness apps, extraverts are likely to be drawn to socially oriented affordances that involve sharing and interacting with others. We hypothesize (H5) that extraverted Fitbit users will actualize affordances such as self-presentation, social recognition, watching others, and updating.

## Methods

### Study Design

This study used a cross-sectional, web-based survey design, and its findings were reported in accordance with the CHERRIES (Checklist for Reporting Results of Internet E-Surveys) checklist [44]. The survey aimed to investigate how users’ personality traits influence their actualization of fitness app affordances. The instrument included validated measures drawn from prior research, covering fitness app affordances and personality traits [7,8,35]. A pilot study was conducted prior to the main data collection to validate the survey instrument. Fitbit, identified as the most downloaded fitness app [36], was selected as the focal platform, and current and previous Fitbit users were recruited as participants.

### Measures

To evaluate the research hypotheses, we adopted the measures and scales from previous information systems, mobile health and fitness app studies that call attention to fitness app affordances [7,8] along with a widely used short version (20 items) measure of the Big Five personality traits [35]. The survey instrument consisted of 4 sections: (1) Big Five personality traits questions (4 items for each trait) [35], (2) 11 fitness app affordances questions (4 items for each affordance) [7,8], (3) user usage status, and (4) demographic information. All items were measured on a 5-point Likert scale with relevant anchors, as shown in MultimediaAppendices 1  2 (the latter was adapted from [9,10]).

### Participants and Recruitment Procedures

Prior to the main study, a pilot study was conducted with participants recruited between June 1 and July 5, 2022 via Sona system participant pool at a university and via social media platforms (Facebook [Meta Platforms Inc] and Reddit [Reddit Inc]). A total of 403 participants completed the pilot study. After excluding incomplete responses, those failing validation checks, or showing signs of inattentive responding (eg, straightlining or unusually short completion time), 81 valid responses remained. These 81 participants were between 18‐52 years old, with 79% (64/81) of the sample 25 years or older. All scales had acceptable reliability with a Cronbach α of at least 0.651, as shown in Multimedia Appendix 3 [45]. The pilot study helped us validate the survey instrument and identified recruitment challenges that were addressed to improve the main study.

For the main study, the target sample size was calculated based on the formula suggested by Gaskin [46]. Given the limitations of social media recruitment (eg, large percentage of invalid responses) identified during the pilot study, for the main study, we recruited participants through Prolific (Prolific Academic Ltd), a crowdsourcing platform known for high-quality data [47]. We first distributed a prescreening survey to 1300 participants to identify current adult US-based Fitbit app users engaging in exercise. We identified 622 users who were then invited to participate in the main study. A total of 506 participants completed the survey. After removing the responses of participants who failed attention checks, 442 valid responses (71%) remained.

### Ethical Considerations

Both the pilot and main studies received approval from the Institutional Review Board (IRB-22‐0351) of Worcester Polytechnic Institute and were conducted in accordance with ethical guidelines for human participant research. Participants were provided informed consent electronically at the beginning of each survey, where they were informed about the study’s purpose, the voluntary nature of participation, and their right to withdraw at any time. The consent form also noted that data would be stored securely and accessed only by the research team. All responses were collected anonymously.

For the pilot study, student participants recruited through the university’s local participant pool received 0.5 course credit as an incentive, whereas participants recruited through social media received an opportunity to enter a raffle for one of 10 US $10 prizes as an incentive. The distribution of incentives was managed by one of the authors. For the main study, all recruitment and distribution of incentives were managed by Prolific. Participants recruited for the prescreening survey received a flat rate of US $0.18 as an incentive. Among these participants, those who were eligible to participate in the actual study (current Fitbit users who engage in exercise) received a flat rate of US $6 as an additional incentive.

## Results

### Descriptive Statistics

The main study sample demographics are illustrated in Table 1. The study sample was balanced by gender (50% male, 48% female, and 2% transgender and nonbinary). Most of the participants were between the ages of 25 and 44 (64%), White (81%), and employed (84%).

**Table 1.** Demographics of the main study sample (N=442), based on an online survey of US-based Fitbit users engaging in regular exercise; source: authors’ own work.

	Participants, % (N=442)
Gender	
Men	50
Women	48
Transgender	1
Nonbinary	1
Age (years)	
18‐24	7
25‐34	33
35‐44	31
45‐54	13
55‐64	11
65‐74	4
75 or older	1
Ethnicity	
White	81
Black	9
Asian	8
Other	2
Employment status	
Full-time	70
Part-time	14
Not in paid work (eg, homemaker, retired, or disabled)	9
Unemployed (and seeking a job)	4
Other	3

All participants were current Fitbit users with varying usage patterns as illustrated in Table 2. Among our sample, 74% (327/442) had used the app for more than a year, 80% (354/442) had tried premium subscription, 44% (194/442) used the app daily, and only 4% (18/442) used the app once a week or less. Only 41% (181/442) reported using Fitbit wearable device. Walking was the most commonly engaged activity tracked by the Fitbit app (274/442, 62%), followed by running (106/442, 24%) and biking (22/442, 5%).

**Table 2.** Usage patterns of the main study sample (N=442), based on an online survey of US-based Fitbit users engaging in regular exercise; source: authors’ own work.

	Participants, % (N=442)
Frequency of Fitbit app use	
Daily	44
4‐6 times a week	31
2‐3 times a week	21
Once a week	2
Less than once a week	2
Duration of Fitbit app use	
Less than 1 month	0
Between 1 week and 1 month	0
1‐3 months	4
4‐6 months	9
7‐9 months	5
10‐12 months	8
More than 12 months	74
Duration of premium use	
Never	20
Up to 3 months or free trial	25
4‐6 months	10
7‐12 months	13
More than 12 months	32
Device used	
Phone	40
Fitbit wearable device	41
Smart watch (such as a Samsung watch and Apple Watch)	11
Laptop/desktop computer	8
Exercise engaged in	
Running	24
Walking	62
Biking	5
Swimming	1
Spinning	1
Hiking	3
Yoga	1
Other	3

### Measurement Model Evaluation

To ensure that the observed variables in our survey accurately represent the underlying constructs in our model, we used SmartPLS 4 software (SmartPLS GmbH) and confirmatory factor analysis to assess internal reliability, convergent validity, discriminant validity, outer loadings, and model fit. As illustrated in Table 3, internal reliability was assessed using Cronbach α, with values above 0.8 indicating satisfactory reliability [45]. Convergent validity was measured using the average variance extracted (AVE), with acceptable values being 0.5 or higher [45]. Outer loadings results exceeded 0.7, confirming their contribution to their constructs. A few outer loadings were slightly lower (0.59 and above), but these were considered acceptable, especially considering the adequate scores of Cronbach α and AVE [45]. Thus, all items were retained in the model.

**Table 3.** Construct reliability, validity measures (Cronbach α and average variance extracted), and factor loadings for the main study constructs; source: authors’ own work.

	Cronbach α	AVE^a^	Item loading				
Construct			1	2	3	4	5
Comparing (Affordance)	0.977	0.894	0.950	0.964	0.969	0.927	0.917
Competing (Affordance)	0.974	0.907	0.914	0.967	0.978	0.949	—^b^
Encourage (Affordance)	0.972	0.897	0.948	0.959	0.927	0.954	—
Guidance (Affordance)	0.911	0.728	0.889	0.924	0.857	0.730	—
Self-presentation (Affordance)	0.973	0.899	0.916	0.953	0.966	0.957	—
Recognize (Affordance)	0.977	0.914	0.941	0.952	0.955	0.975	—
Reminding (Affordance)	0.973	0.901	0.926	0.934	0.968	0.969	—
Rewards (Affordance)	0.965	0.861	0.877	0.894	0.969	0.968	—
Searching (Affordance)	0.940	0.798	0.885	0.879	0.908	0.901	—
Updating (Affordance)	0.857	0.610	0.681	0.714	0.884	0.827	—
Watching others (Affordance)	0.961	0.864	0.936	0.957	0.945	0.877	—
Agreeableness (Big Five)	0.870	0.619	0.882	0.844	0.687	0.717	—
Conscientiousness (Big Five)	0.843	0.584	0.675	0.595	0.887	0.861	—
Emotional stability (Big Five)	0.828	0.552	0.701	0.626	0.870	0.753	—
Extraversion (Big Five)	0.913	0.726	0.778	0.870	0.881	0.874	—
Openness (Big Five)	0.826	0.548	0.631	0.773	0.762	0.784	—

a AVE: average variance extracted.

b Not applicable.

Discriminant validity was evaluated using the Fornell and Larcker [48] criterion. The results, illustrated in Table 4, showed that the AVE values of each construct were higher than the correlations with other constructs, indicating satisfactory discriminant validity. Model fit indices also supported a good model fit: the comparative fit index was 0.929, Tucker-Lewis index was 0.922, root mean square error of approximation was 0.054, and standardized root mean square residual was 0.051, all within acceptable ranges [45].

**Table 4.** Discriminant validity assessment using the Fornell-Larcker criterion for main study constructs; source: authors’ own work.

Construct	Agree	Compare	Compete	Cons	Extra	Emot	Openn	Present	Remind	Search	Encourage	Guidance	Recognize	Reward	Update	Watch
Agree	0.787	0.091	0.036	0.003	0.222	0.072	0.322	0.057	0.107	0.122	0.103	0.145	0.082	0.051	0.145	0.07
Compare	0.091	0.946	0.786	−0.041	0.201	−0.017	0.012	0.523	0.251	0.383	0.701	0.419	0.662	0.498	0.153	0.791
Compete	0.036	0.786	0.952	−0.061	0.181	−0.011	−0.004	0.512	0.161	0.388	0.764	0.38	0.716	0.535	0.099	0.797
Cons	0.003	−0.041	−0.061	0.764	0.003	0.401	0.135	0.064	0.017	0.137	−0.069	0.029	−0.064	−0.095	0.16	−0.053
Extra	0.222	0.201	0.181	0.003	0.852	0.27	0.237	0.138	0.05	0.072	0.206	0.124	0.167	0.079	0.023	0.147
Emot	0.072	−0.017	−0.011	0.401	0.27	0.743	0.208	0.05	−0.057	0.058	−0.046	0.077	−0.037	−0.051	0.049	−0.058
Openn	0.322	0.012	−0.004	0.135	0.237	0.208	0.74	0.016	0.066	0.074	−0.013	0.024	0.02	−0.046	0.2	0.001
Present	0.057	0.523	0.512	0.064	0.138	0.05	0.016	0.948	0.259	0.362	0.57	0.436	0.587	0.506	0.2	0.528
Remind	0.107	0.251	0.161	0.017	0.05	−0.057	0.066	0.259	0.949	0.297	0.316	0.405	0.256	0.28	0.443	0.258
Search	0.122	0.383	0.388	0.137	0.072	0.058	0.074	0.362	0.297	0.893	0.423	0.745	0.421	0.371	0.38	0.49
Encourage	0.103	0.701	0.764	−0.069	0.206	−0.046	−0.013	0.57	0.316	0.423	0.947	0.473	0.848	0.564	0.195	0.771
Guidance	0.145	0.419	0.38	0.029	0.124	0.077	0.024	0.436	0.405	0.745	0.473	0.853	0.443	0.451	0.386	0.507
Recognize	0.082	0.662	0.716	−0.064	0.167	−0.037	0.02	0.587	0.256	0.421	0.848	0.443	0.956	0.557	0.126	0.727
Reward	0.051	0.498	0.535	−0.095	0.079	−0.051	−0.046	0.506	0.28	0.371	0.564	0.451	0.557	0.928	0.119	0.548
Update	0.145	0.153	0.099	0.16	0.023	0.049	0.2	0.2	0.443	0.38	0.195	0.386	0.126	0.119	0.781	0.15
Watch	0.07	0.791	0.797	−0.053	0.147	−0.058	0.001	0.528	0.258	0.49	0.771	0.507	0.727	0.548	0.15	0.929

### Hypothesis Testing

The model was initially evaluated using the coefficient of determination (*R*²) to assess how well the independent variables explained the variance in different affordances related to fitness app usage. The *R*² values indicated that the model explained a small to moderate amount of variance across the different affordances: 1.7% for comparing, 0.5% for competing, 1.8% for encouragement, 3.1% for guidance, 3.5% for recognition, 0.7% for reminding, 1.0% for rewards, 0.8% for searching, 2.4% for self-presentation, 6.3% for updates, and 3.3% for watching others. These findings suggest that the included variables provide limited explanatory power for these constructs, highlighting the need for further exploration of additional factors that might influence user interaction with fitness apps.

**Figure 1.**
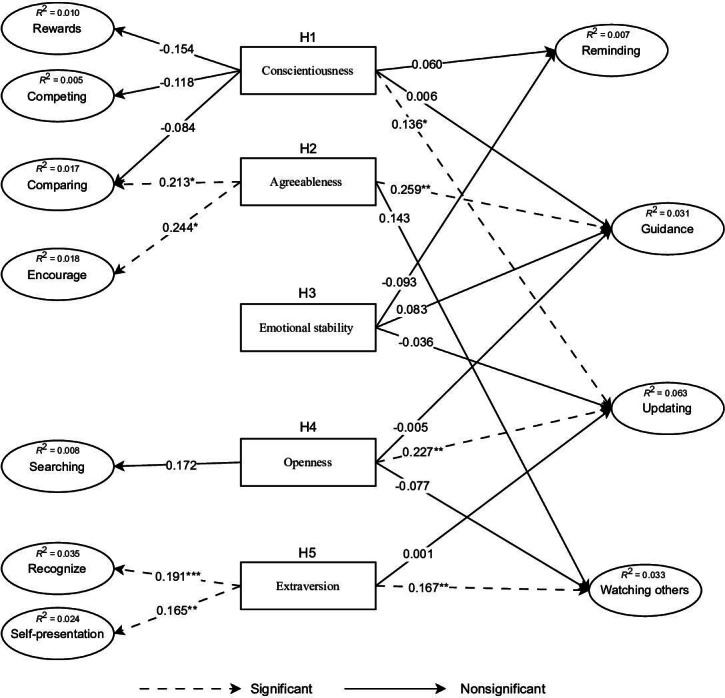
Study model results showing the relationships between Big Five personality traits and fitness app affordance actualizations; source: authors’ own work.

The results are presented in detail in Figure 1.

Conscientiousness significantly influenced updates (β=0.136, *P*=.01). The influence on rewards was not. Likewise, conscientiousness did not significantly influence competing, comparing, reminding, or guidance. While the hypotheses suggested a positive relationship between conscientiousness and these affordances, only update was shown to have a significant positive impact. Agreeableness had significant positive effects on guidance (β=0.259, *P*=.003), encouragement (β=0.244, *P*=.01), and comparing (β=0.213, *P*=.02). However, its effect on watching others was not significant. The significant positive results support the hypothesized relationship between agreeableness and the actualization of these affordances. Emotional stability has a nonsignificant effect on guidance, reminding, and updates. The results reject the hypothesized positive relationship between low emotional stability and actualizing these affordances. Openness had a significant positive effect on updates (β=0.227, *P*=.001). However, it did not significantly influence searching, watching others, or guidance. The results support the study hypothesis on the relationship between openness and actualizing update affordances and reject all the other hypotheses on the relationship between openness and actualizing the affordances. Extraversion showed significant positive effects on recognize (β=0.191, *P*<.001), self-presentation (β=0.165, *P*=.002), and watching others (β=0.167, *P*=.003). However, the effect on updates was not significant. The results support the study hypothesis on the relationship between extraversion and actualizing these affordances, except for the update affordance. The results are presented in Table 5.

**Table 5.** Results for the hypotheses; source: authors’ own work.

Hypothesis^a^	Path coefficient	*P* value	Hypothesis support
H1: Conscientiousness – Guidance	0.006	0.98	Not supported
H1: Conscientiousness – Reminding	0.060	0.44	Not supported
H1: Conscientiousness – Updating	0.136	0.01	Supported
H1: Conscientiousness – Competing	−0.118	0.18	Not supported
H1: Conscientiousness – Comparing	−0.084	0.33	Not supported
H1: Conscientiousness – Rewards	−0.154	0.06	Not supported
H2: Agreeableness – Comparing	0.213	0.02	Supported
H2: Agreeableness – Guidance	0.259	0.003	Supported
H2: Agreeableness – Watching others	0.143	0.13	Not supported
H2: Agreeableness – Encourage	0.244	0.01	Supported
H3: Emotional Stability – Updating	−0.036	0.49	Not supported
H3: Emotional stability – Guidance	−0.083	0.30	Not supported
H3: Emotional stability – Reminding	−0.093	0.29	Not supported
H4: Openness – Searching	0.172	0.11	Not supported
H4: Openness – Watching others	−0.077	0.50	Not supported
H4: Openness – Guidance	−0.005	0.96	Not supported
H4: Openness – Updating	0.227	0.001	Supported
H5: Extraversion – Self-presentation	0.165	0.002	Supported
H5: Extraversion – Recognize	0.191	0.000	Supported
H5: Extraversion – Watching others	0.167	0.003	Supported
H5: Extraversion – Updating	0.001	0.98	Not supported

a Model fit: CFI=.929, TLI=.922, RSMEA=.054, SRMR=.051*.*

## Discussion

### Principal Findings

Based on the affordance actualization theory [12,49] and empirical research on the impact of personality traits on behavior [25,38], this study suggests that users’ personality traits play a role in the actualization of affordances. The findings of the study support the connection between personality traits and affordance actualization, providing valuable insights. While not all predicted relationships were confirmed, each personality trait influenced the actualization of specific affordances, confirming some of the hypotheses. Specifically, agreeableness has a positive impact on the actualization of comparing, guidance, and encouragement affordances; extraversion influences recognition, self-presentation, and watching others; and conscientiousness and openness affect updating.

### Theoretical Contribution

This research contributes to the existing literature on affordance theory. Previous studies have found that people’s perceptions and actualizations of an object’s affordances are influenced by factors such as ability, age, social context, preference, situation, policies, procedures, and rules [10,12,50]. Furthermore, this study emphasizes the role of personality traits as a crucial factor that impacts users’ perceptions and actualizations of affordances.

This study also contributes to the literature on user interaction with technology functionality and design [19,51]. The findings highlight that personality traits extend beyond technology functionality to affect how users perceive and actualize what these technologies afford to them. In personality research, conscientious individuals are described as organized, efficient, and goal-oriented [21]. Previous studies have shown that conscientious individuals are more likely to use technologies that support task completion and collaboration, especially when these technologies provide reminders to perform specific actions [15,22]. Thus, conscientious users will be more likely to actualize affordances that support goal achievement and self-monitoring, such as updating, guidance, reminding, competing, comparing, and rewards. Our results indicated that among the hypothesized affordances, only updating was significantly actualized by conscientious users. Conscientious users actualize updating affordances using Fitbit to stay on track with their fitness goals, reflecting their organized and disciplined nature.

The absence of actualization of other affordances among conscientious users could be due to user preferences for specific Fitbit features. For example, some users may prefer alternative features to actualize guidance instead of the features offered by Fitbit. Therefore, focusing solely on Fitbit may limit the variety of features that some users prefer over those provided by Fitbit to actualize these affordances. Individuals who score high in agreeableness are described as being cooperative, considerate, and good-natured [21]. Previous research suggests that agreeable individuals are more likely to use technologies that support collaboration and social interaction [25]. For instance, agreeable students find university bulletin boards and collaborative classroom systems especially useful [25,26]. Moreover, a study on gamification apps indicated a positive correlation between agreeableness and engagement in challenges with others [19]. In the context of fitness apps, we predict that agreeable users will actualize affordances that promote social interaction and collaboration. Our study corroborates these findings, demonstrating that agreeable users effectively actualize comparison, guidance, and encouragement affordances, underscoring their inclination toward seeking and appreciating social interactions and collaborative activities in their fitness app use.

People who are high in openness to experience tend to be imaginative, curious, and open-minded [21]. Studies suggest that individuals with high levels of openness are more likely to use authoritative strategies to achieve their fitness goals [23].

Our research indicates that openness affects the actualization of updating affordance in fitness apps, but not guidance affordance. This aligns partly with existing literature showing that open individuals prefer helpful strategies but may not necessarily seek guidance. We also expected that people high in openness would be more inclined to seek new information to improve their fitness. However, our results did not support this assumption. The lack of interest in seeking new information may be due to how this feature is presented. Often, the available information is shared by users in posts, which, although useful, may lack reliable sources, making the information less trustworthy despite its potential accuracy.

Individuals high in extraversion are sociable, outgoing, and energetic [21]. Previous research suggests that people who are extraverted and use social media platforms such as Facebook tend to be more socially active. They frequently upload photos, update their statuses, and have larger social networks [42]. Their social nature motivates them to continue using social media [43]. Based on this, we hypothesized that extraversion has a positive influence on social aspects of apps, such as recognition, self-presentation, and observing others’ actions in fitness apps. Our results support this hypothesis by indicating that extraverted users are likely to seek social interaction and recognition, using the app to share their achievements and follow others’ activities.

People with low emotional stability are prone to stress, anxiety, and emotional fluctuations [21]. Previous studies have found that these individuals are less likely to accept and actively use technology. In this study, we focused on current Fitbit users who have voluntarily accepted and used the device, likely motivated by health concerns. Therefore, we hypothesized that these users are primarily driven by health improvement rather than curiosity, enjoyment, collaboration, or external motivations. We expected them to use fitness app features related to health, such as updates, guidance, and reminders, to monitor their health status, perform exercises correctly, and maintain regular exercise routines. However, our results did not show any influence on the expected features. This lack of significant findings might be due to the characteristics of our study sample. A homogeneous sample with slight variation in personality traits may not reveal significant relationships, whereas a more diverse sample might highlight stronger influences.

### Practical Contribution

The study findings also have practical implications for the design of fitness apps. Since personality traits influence users’ actualization of an app’s affordances, it becomes imperative to consider the potential implications of user personality traits when designing and implementing affordances in fitness apps. Considering the results, removing certain features that facilitate actualizing some undesirable affordances for users with particular personality traits, such as conscientious users, could be a viable strategy to prevent the possible negative actualization of some affordances. For example, the challenges feature in the Fitbit app can play a prominent role in actualizing reward affordances. By excluding this feature for users with the conscientiousness traits, conscientious users would not be bothered with less-needed affordances.

### Limitation and Future Research

This study is focused on characterizing users based on the Big Five personality traits, to gain an abstract understanding of users’ motives in using specific affordances. Future research could expand user characterization by considering additional factors such as culture, economy, situation, circumstances, age, and gender to achieve a more precise understanding of how users interact with fitness apps.

The study primarily examined the context of Fitbit; however, it is important to acknowledge that different fitness apps may have distinct designs and feature sets. These differences in technology design can influence users’ perceptions of technology affordances. Therefore, future research should explore a broader range of fitness apps, considering the differences in their technological designs and assessing how these variations impact users’ actions.

### Conclusion

This study investigates the influence of users’ personality traits on affordance actualization within the context of fitness apps. It highlights how users’ characterizations (personality traits) shape their interactions with technology, leading them to actualize various affordances based on these traits. These findings enhance our understanding of the factors impacting IT interaction and provide valuable insights for designing user-centered technologies that promote greater user interaction.

## Supplementary material

10.2196/72691 Multimedia Appendix 1 Survey items used to measure Big Five personality traits using the prompt “Indicate how accurately each statement describes you” and responses on a 5-point Likert Scale (1=very inaccurate, 5=very accurate); source: authors’ own work.

10.2196/72691 Multimedia Appendix 2 Survey items used to measure fitness app affordances using the prompt “I use Fitbit for [user’s selected exercise, eg, walking, is inserted here] to” and responses on a 5-point Likert Scale (1=strongly disagree to 5=strongly agree); source: authors’ own work.

10.2196/72691 Multimedia Appendix 3 Cronbach α scores for the pilot study; source: authors’ own work.

## Abbreviations

AVE: average variance extracted
CHERRIES: Checklist for Reporting Results of Internet E-Surveys
