# Supplementary material for: Personality-Driven Variations in Fitness App Affordance Actualization Among Adults: Quantitative Survey Study
Source: JMIR Form Res. 2025 Sep 12;9:e72691. doi: 10.2196/72691 (PMC12431158; doi:10.2196/72691)
Supplement: Multimedia Appendix 1 [file formative-v9-e72691-s001.pdf]

Construct	Question
Extraversion	I am the life of the party
	I talk to a lot of different people at parties
	I don't talk a lot
	I keep in the background
Agreeableness	I sympathize with others' feelings
	I feel others' emotions
	I am not interested in other people's problems
	I am not really interested in others
Conscientiousness	I get chores done right away
	I like order
	I make a mess of things
	I often forget to put things back in their proper place
Emotional Stability	I am relaxed most of the time
	I seldom feel blue
	I get stressed out easily
	I have frequent mood swings
Openness	I have a vivid imagination
	I have difficulty understanding abstract ideas
	I am not interested in abstract ideas
	I do not have a good imagination
