# Supplementary material for: Personality-Driven Variations in Fitness App Affordance Actualization Among Adults: Quantitative Survey Study
Source: JMIR Form Res. 2025 Sep 12;9:e72691. doi: 10.2196/72691 (PMC12431158; doi:10.2196/72691)
Supplement: Multimedia Appendix 2 [file formative-v9-e72691-s002.pdf]

Construct	Question
Comparing	Compare my exercise activities to other people’s exercise activities
	See how other people’s exercise activities compare to mine
	Compare my exercise activities to the exercise activities of others
	Track my exercise activities with respect to how they compare to others
	Rank my exercise activities relative to others’ exercise activities
Competing	Compete with other people
	Challenge other individuals to exercise competitions
	Have exercise contests with other individuals
	Enter into exercise competitions with others
Encourage	Have other people encourage my exercise activities
	Receive encouraging messages regarding my exercise activities from others
	Have my exercise accomplishments acknowledged by other people
	Receive moral support for my exercise activities from others
Guidance	Get guidance how to better perform physical exercise
	Get taught how to improve my exercise activity
	Receive instructions while doing exercise activity
	Get supervised to reach my exercise activity goals
Self-presentation	Express myself as a physically active person
	Establish a preferred image of myself as physically active person
	Present myself as physically active person
	Project an image about myself as physically active person
Recognize	Earn compliments from others for my exercise activity
	Earn respect of others for my exercise activity
	Get recognition from others for my exercise behavior
	Get noticed by others for my exercise activity
Reminding	Remind me to do an exercise activity
	Notify me to perform an exercise activity
	Provide me with reminders when I need to do an exercise activity
	Prompt me when I need to perform an exercise activity
Rewards	Receive rewards for my exercise activities
	Obtain rewards for my exercise activities
	Win prizes for my exercise activities
	Earn prizes for my exercise activities
Searching	Search for exercise information
	Access exercise information
	Find exercise information that is relevant to me
	Browse exercise information
Updating	Provide me with messages about my exercise progress
	Give me visual cues about my exercise progress
	Provide me with exercise progress updates
	Update me with the status of my exercise progress
Watching others	Observe others who are performing exercise activities
	Follow exercise activities of other people
	Keep an eye on other people’s way of doing exercise
	Get inspired by how others do exercise activity
