# Supplementary material for: Personality-Driven Variations in Fitness App Affordance Actualization Among Adults: Quantitative Survey Study
Source: JMIR Form Res. 2025 Sep 12;9:e72691. doi: 10.2196/72691 (PMC12431158; doi:10.2196/72691)
Supplement: Multimedia Appendix 3 [file formative-v9-e72691-s003.pdf]

**Fitness app affordances**	**Cronbach's Alpha (N=81)**
Comparing	.900
Competing	.975
Reminding	.887
Searching	.882
Encouraging	.888
Guidance	.899
Recognizing	.908
Reward	.920
Self-presentation	.867
Updating	.847
Watching others	.908
**Personality Traits**	
Openness to experience	.742
Emotional stability	.677
Agreeableness	.799
Extraversion	.651
Conscientiousness	.780
